# Complete Genome Sequences of Paenibacillus larvae Phages Halcyone, Heath, Scottie, and Unity from Las Vegas, Nevada

**DOI:** 10.1128/MRA.00977-18

**Published:** 2018-09-27

**Authors:** Diane G. Yost, Carolyn Chang, Lucy LeBlanc, Erin Cassin, Ceara Peterman, Padmani Rai, Alicia Salisbury, Nicolas Barroga, Ramiro Cisneros, Joseph Fersini, Jonathan Juste, Juvie Ines, Gabriel Leyva, Dyanne Macalinao, Spencer Muscelli, Gustavo S. Reyes, Heather Rhoden, Rodney Tan, Erika Torres, Krystal Tran, Georgette Uriarte-Valle, Christopher Wallace, Simon Wong, Kevin Ayala-Pineda, Vanessa Cadiz, Tiffany Jeanite, Sophia Nhan, Julianne H. Grose, Christy Strong, Penny S. Amy, Philippos K. Tsourkas

**Affiliations:** a School of Life Sciences, University of Nevada, Las Vegas, Nevada, USA; b Department of Microbiology and Molecular Biology, Brigham Young University, Provo, Utah, USA; University of Maryland School of Medicine

## Abstract

We present the complete genome sequences of four phages that infect Paenibacillus larvae, the causative agent of American foulbrood disease in honeybees. The phages were isolated from beehives and beeswax products from Las Vegas, Nevada. The genomes are 50 to 55 kbp long and use the “direct terminal repeats” DNA-packaging strategy.

## Abstract (precis)

We present the complete genome sequences of four phages that infect Paenibacillus larvae, the causative agent of American foulbrood disease in honeybees. The phages were isolated from beehives and beeswax products from Las Vegas, Nevada.

## ANNOUNCEMENT

American foulbrood, currently the most destructive bacterial disease affecting the honeybee, Apis mellifera, is caused by the Gram-positive bacterium Paenibacillus larvae (1). There is growing interest in phages that infect and lyse P. larvae, as antibiotic resistance is now widespread (2). There are currently 26 complete P. larvae phage genome sequences in the literature and more in the process of being published (3–8). Here, we present the complete genome sequences of phages Halcyone and Heath, isolated from soil underneath healthy hives in Gilcrease Orchards in North Las Vegas, Nevada; phage Unity, isolated from material inside a beehive at the University of Nevada Las Vegas (UNLV); and phage Scottie, isolated from commercial hand cream (Burt’s Bees) purchased in the Las Vegas area.

The phages were amplified using P. larvae NRRL 2605, an ERIC I genotype strain, and plated on modified brain heart infusion agar with soft agar. Phage DNA was purified with phenol-chloroform extraction at the University of Nevada Las Vegas (UNLV) and sequenced at Brigham Young University with Illumina HiSeq 2500 sequencing with 250-bp paired-end reads. The genome sequences were assembled with Geneious v. 10.2.2 (Biomatters, Auckland, New Zealand) with medium-low sensitivity/fast and manually annotated using DNA Master (9) by students in the course BIOL 209X Phage Discovery at UNLV.

Each phage's GenBank accession number, isolation source, and assembly results are shown in Table 1. All 4 phages are in the family *Siphoviridae* with linear double-stranded DNA (dsDNA) genomes and use the “direct terminal repeats” (DTR) DNA-packaging strategy (10, 11). The DTR sequence of each phage was identified using Pile-up Analysis Using Starts & Ends (PAUSE) (https://cpt.tamu.edu/computer-resources/pause/) and Geneious, looking for a sharply delimited region with double coverage depth (11). Halcyone, Heath, and Scottie have identical DTR sequences 377 bp long, whereas Unity has a different DTR sequence 378 bp long. The genomes were oriented by setting the first base of the DTR sequence to be the first base of the genome.

**TABLE 1:** GenBank accession numbers and genome assembly results for *Paenibacillus larvae* phages

Phage name	GenBank accession no.	Isolation source	Genome assembly results			
			Genome length (bp)	Avg coverage (×)	GC content (%)	DTR sequence length (bp)
Halcyone	MH460827	Soil	55,560	83	48.6	377
Heath	MH460826	Soil	55,560	222	48.6	377
Scottie	MH460825	Hand cream	55,990	173	48.6	377
Unity	MH460824	Beehive	50,316	320	49.1	378

The genomes for Halcyone, Heath, and Scottie are 55 kbp long, which is at the maximum of the range of the P. larvae phage genome length (3–8), and the genome of Unity is 50 kbp long. A multiple alignment of genome sequences with ClustalW shows that Halcyone and Heath are very closely related to each other, while Scottie is a little more distant and Unity is more distant still; the difference is largely due to a 5-kbp region missing in Unity but present in the other three phages. All four phages encode a large terminase, a portal protein, a major tail protein, two tail assembly proteins, a tail tape measure protein, and an *N*-acetylmuramoyl-l-alanine amidase. The tail assembly proteins appear to have a predicted translational frameshift similar to those of the G and G-T genes in phage Lambda (12, 13), located in the 3′ region of the upstream tail assembly protein (gp14). We tentatively identify the heptanucleotide slippery sequence as “TAAAAAA.” Current work is ongoing to identify more P. larvae phage protein functions and provide a comparative genomic analysis of P. larvae phages.

### Data availability.

The GenBank accession numbers for the four complete Paenibacillus larvae phage genome sequences are listed in Table 1.
